# Molecular mechanisms of endothelial–mesenchymal transition and its pathophysiological feature in cerebrovascular disease

**DOI:** 10.1186/s13578-025-01393-y

**Published:** 2025-04-19

**Authors:** Huimin Jiang, Yifan Zhou, Weiyue Zhang, Hui Li, Wei Ma, Xunming Ji, Chen Zhou

**Affiliations:** 1 Beijing Institute of Brain Disorders, Laboratory of Brain Disorders, Collaborative Innovation Center for Brain Disorders, Beijing Advanced Innovation Center for Big Data-Based Precision Medicine, Ministry of Science and Technology, Capital Medical University, Beijing, 100069 China; 2 Beijing Advanced Innovation Center for Big Data-Based Precision Medicine, School of Biological Science and Medical Engineering, Beihang University, Beijing, 100191 China; 3 Department of Neurology, Xuanwu Hospital, Capital Medical University, Beijing, China

**Keywords:** Cerebrovascular disease, Endothelial cells, EndMT, Vascular remodeling

## Abstract

The phenomenon of endothelial–mesenchymal transition (EndMT), a distinct subtype of epithelial–mesenchymal transition (EMT), has garnered significant attention from scholars. EndMT refers to the process whereby endothelial cells (ECs) transform into mesenchymal cells in response to various stimuli, resulting in the loss of their original characteristics. This process has diverse implications in both physiological and pathological states. Under physiological conditions, EndMT plays a crucial role in the development of the cardiovascular system. Conversely, under pathological conditions, EndMT has been identified as a pivotal factor in the development of cardiovascular diseases. Nonetheless, a comprehensive overview of EndMT in cerebrovascular disease is currently lacking. Here, we discuss the heterogeneity of EndMT occurrence and the regulatory factors involved in its development and analyze the feasibility of EndMT as a therapeutic target, aiming to provide a solid theoretical foundation and evidence to address diseases caused by pathological EndMT.

## Introduction

The most important players in the pathogenesis of cardiovascular diseases are vascular endothelial cells (VECs), which constitute the innermost layer of every blood vessel. Their function is to maintain the normal homeostasis of the cardiovascular system. Studies have shown that ECs possess higher plasticity and heterogeneity, as they have advanced specialized functions in different organs and vascular beds [1, 2]. In response to various stimuli, activated ECs undergo EndMT, progressively and dynamically losing their specific endothelial markers concurrent with acquiring a mesenchymal profile and transforming into mesenchymal cells [3–5]. During this process, the cellular morphology transitions from dense cobblestone-like to spindle-like, resulting in impaired characteristic functions of ECs and enhanced mesenchymal properties, such as invasion and migration. Moreover, EndMT also affects the physiological properties of stem cells [6]. Specifically, ECs undergo a gradual process of dedifferentiation and acquire multipotential properties similar to those of mesenchymal stem cells (MSCs), which eventually differentiate into a variety of cell types, such as fibroblasts/myofibroblasts, osteocytes, chondrocytes, and adipocytes [7].

The EndMT program is orchestrated by various biochemical, biomechanical, and environmental signals initiated by multiple signaling pathways and TFs [8]. ECs exhibit many characteristics similar to those of epithelial cells, including apical–basal polarity, tight cell junctions, and the ability to transition to mesenchymal-like cells. Researchers suggest that EndMT is partially regulated by the same signaling pathways (TGFβ, Wnt, FGF, Notch, miRNA, epigenetic regulation, and histone modification) and key transcription factors [9]. Alterations in these pathways control the expression of transcription factors such as Snail [10, 11], Slug [12], Twist [11], ZEB1/ZEB2 [13], Sox2 [14], and Sox9 [15], which initiate EndMT and convert ECs to a mesenchymal state. In combination with genetic markers of ECs and animal models of disease [16], EndMT has been shown to favor the progression of various diseases, including pulmonary arterial hypertension [17], atherosclerosis [18], fibrosis disease [19–21], and cancer [22].

In this review, we present the process of EndMT development and pluripotency differentiation, analyze and summarize the regulatory network of EndMT, and finally discuss its contribution to cerebrovascular disease. We review the development and regulatory mechanisms of EndMT to better understand its role and provide effective theoretical support for developing practical and effective targeted therapeutic strategies.

## EndMT in different types of ECs

ECs are diverse across different tissues, and not all ECs can undergo EndMT. Ferreira et al. reported that ECs obtained from different regions of the vascular system exhibited different propensities to undergo EndMT, which may be related to the anatomical origin of ECs [23]. Among VECs, aortic valvular ECs, a special type of EC present in heart valves, can be easily prompted to undergo EndMT in response to TGF-β signaling [24–26]. Furthermore, the plasticity of ECs in the endocardium may also increase the propensity of VECs to undergo EndMT [27, 28]. Using an endothelial lineage tracking system, Evrard et al. reported that arterial ECs undergo EndMT to generate fibroblast-like cells driven by TGF-β signaling, oxidative stress, and hypoxia, which contribute to atherosclerotic plaque formation in mice and humans [29]. Studies from Cooley and colleagues showed that ECs contribute to neointimal formation through EndMT activated by TGF-β/Smad2/3-Slug signaling during vein graft remodeling. This phenomenon is also present in murine endothelial hemangioendothelioma cells, murine aortic ECs, and human umbilical vein endothelial cells (HUVECs) [30]. Maleszewska et al. revealed that TGF-β2- and IL-1β-treated HUVECs exhibited EndMT features associated with p65-dependent NF-κB activation [31]. Microvascular intestinal ECs and human dermal microvascular ECs do not undergo EndMT in response to TGF-β alone and exhibit strong EndMT when exposed to TNFα [32]. Lymphatic ECs can also undergo EndMT. Kaposi sarcoma herpesvirus infection of lymphatic ECs stimulates EndMT through viral protein activation of the Notch pathway and membrane-type-1 matrix metalloproteinase [33]. At present, the molecular basis for the differential sensitivity of valve, arterial, venous, microvascular, and lymphatic VECs to EndMT signaling is not fully understood.

**Fig. 1:**
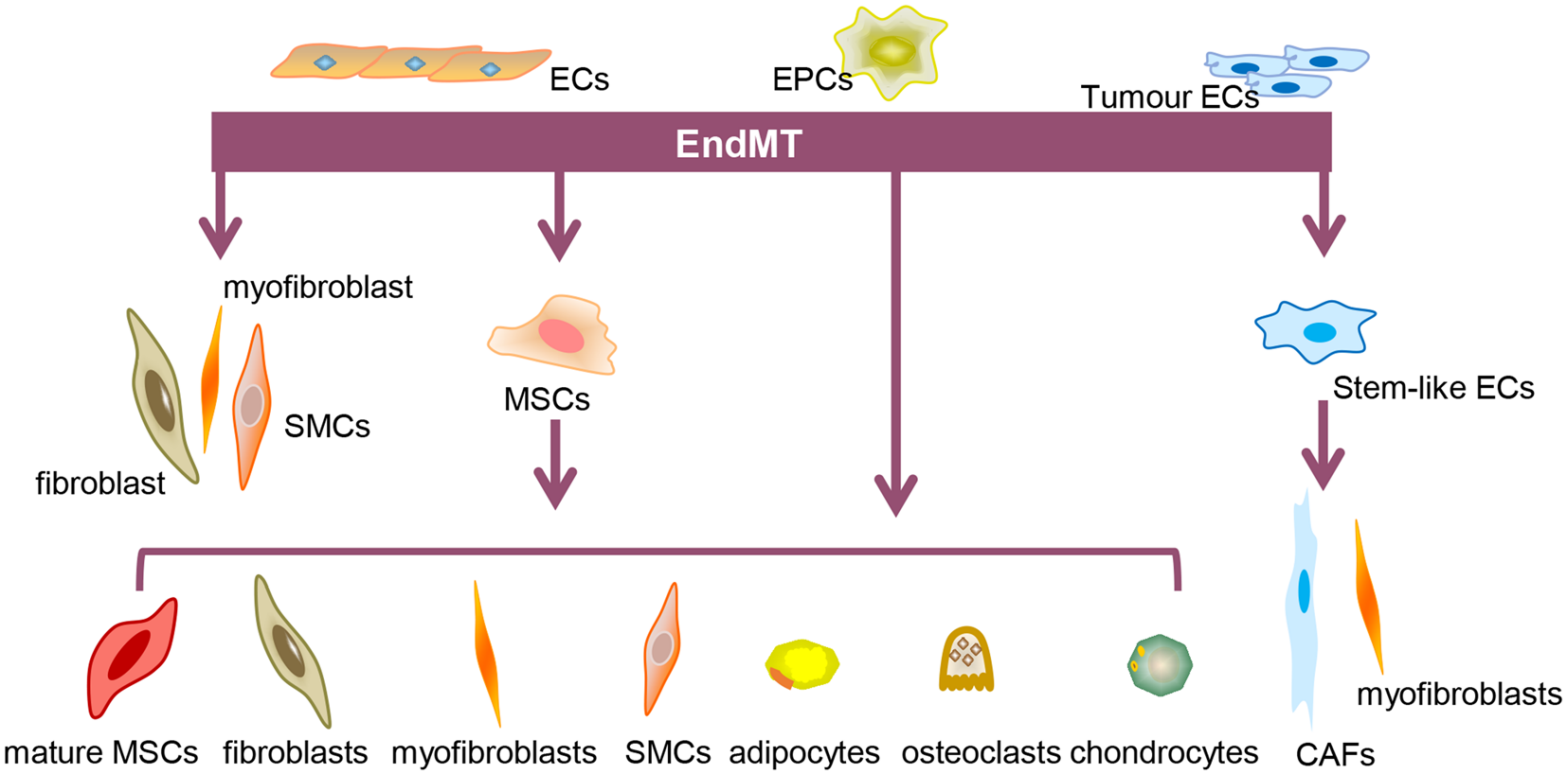
Different types of EndMT. The first type of EndMT is the direct transdifferentiation of ECs into fibroblasts, myofibroblasts, and SMCs, among other types of cells. Second, ECs lose their properties and transform into MSCs, which in turn differentiate into a variety of cell types, such as mature mesenchymal cells, fibroblasts, myofibroblasts, SMCs, adipocytes, osteoblasts, and chondrocytes. The third type consists of EPCs that can be transdifferentiated into mesenchymal cells under specific conditions and then undergo transformation similar to the second type. The fourth type consists of tumor ECs specific to the tumor tissue and can be transdifferentiated into stem cell-like cells and then into cancer-associated fibroblasts (CAFs) and myofibroblasts

There are different ways to induce EndMT depending on the type of ECs. As shown in Fig. 1, the most common type is the conversion of ECs into fibroblasts, myofibroblasts, or smooth muscle cells (SMCs), frequently observed in cardiac development, cardiac fibrosis, and other diseases. In the second type of EndMT, ECs first transform into MSCs and then differentiate into mature mesenchymal cells, including fibroblasts, myofibroblasts, SMCs, mural cells, osteoblasts, chondrocytes, or adipocytes [6]. In the third type of EndMT, endothelial progenitor cells (EPCs) are directly transformed into mesenchymal cells in a specific context. In the pulmonary arteries of patients with chronic obstructive pulmonary disease, bone marrow-derived EPCs infiltrate the intima and differentiate into SMCs [34]. In addition, increased expression of EndMT-associated transcription factors Snail, Slug, Twist, and ZEB1 indicates the transition of EPCs to mesenchymal cells via EndMT [35]. In the fourth type of EndMT, tumor ECs acquire some characteristics of stem cells through EndMT and transform into tumor-associated fibroblasts. This process is accompanied by high secretion of TGF-β, which promotes the mesenchymal transformation of tumor ECs and accelerates the development of tumor metastasis [36].

**Fig. 2:**
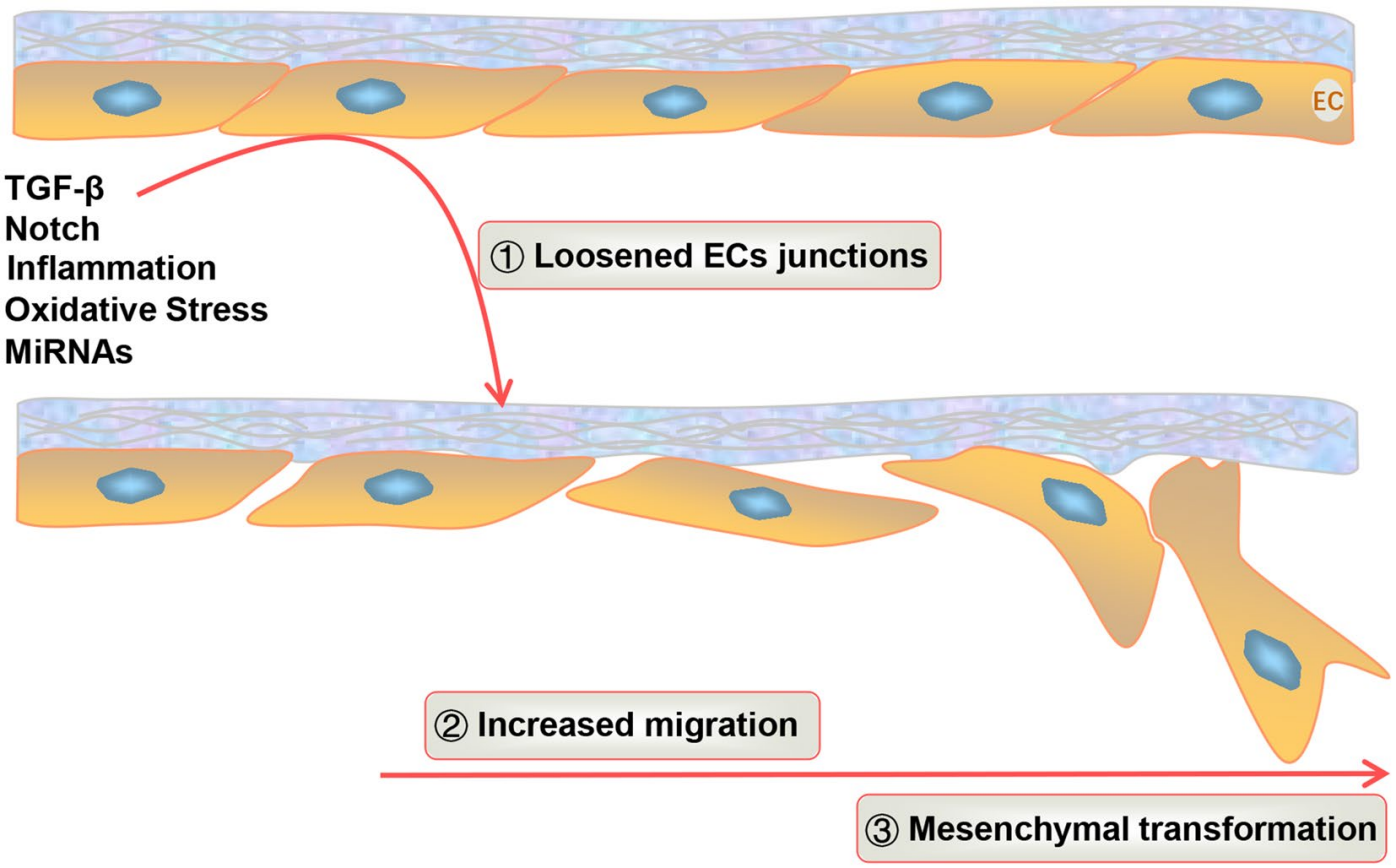
ECs initiate EndMT by loosening ECs junctions under multiple factors. TGF-β, inflammatory factors, and other stimuli initiate EndMT in ECs. In the initiation phase, EC junctions loosen, enabling cell migration to specific sites. The mesenchymal transition phase can occur in two forms: a transitional phase in which cells exhibit both endothelial and mesenchymal characteristics and another where cells fully acquire mesenchymal cell characteristics

Regardless of the EC type, ECs capable of undergoing EndMT are characterized by a phenotypic switch that includes (1) the loss of cell adhesion due to the downregulation of proteins involved in cell‒cell junctions [37]; (2) the transformation of tightly packed, dense cobblestone-like cells into spindle-shaped cells that lack apical‒basal polarity [16]; (3) decreased expression of different ECs markers, such as VE-cadherin, platelet‒endothelial cell adhesion molecule (PECAM-1/CD31), Tie1, Tie2, and von Willebrand factor; and (4) increased expression of mesenchymal cell markers, such as FSP-1, α-SMA, vimentin, and Slug [15, 38, 39]. As shown in Fig. 2, when ECs are exposed to stimulatory factors such as TGF-β, the tight junctions between cells become loosened, promoting ECs migration to the perivascular compartment and transformation to mesenchymal cells, completing the EndMT process.

Different cell fates are revealed when cells switch to a mesenchymal phenotype during EndMT, and cells may exhibit both endothelial and mesenchymal characteristics. These transitional cells may play a critical role in tissue repair but tend to exacerbate fibrosis in the context of disease. ECs that undergo EndMT may also revert to a functional endothelial phenotype and still play a barrier role; they may also exhibit a robust mesenchymal phenotype and perform essential functions in cardiovascular development and vascular remodeling.

## Regulatory factors of EndMT

**Fig. 3:**
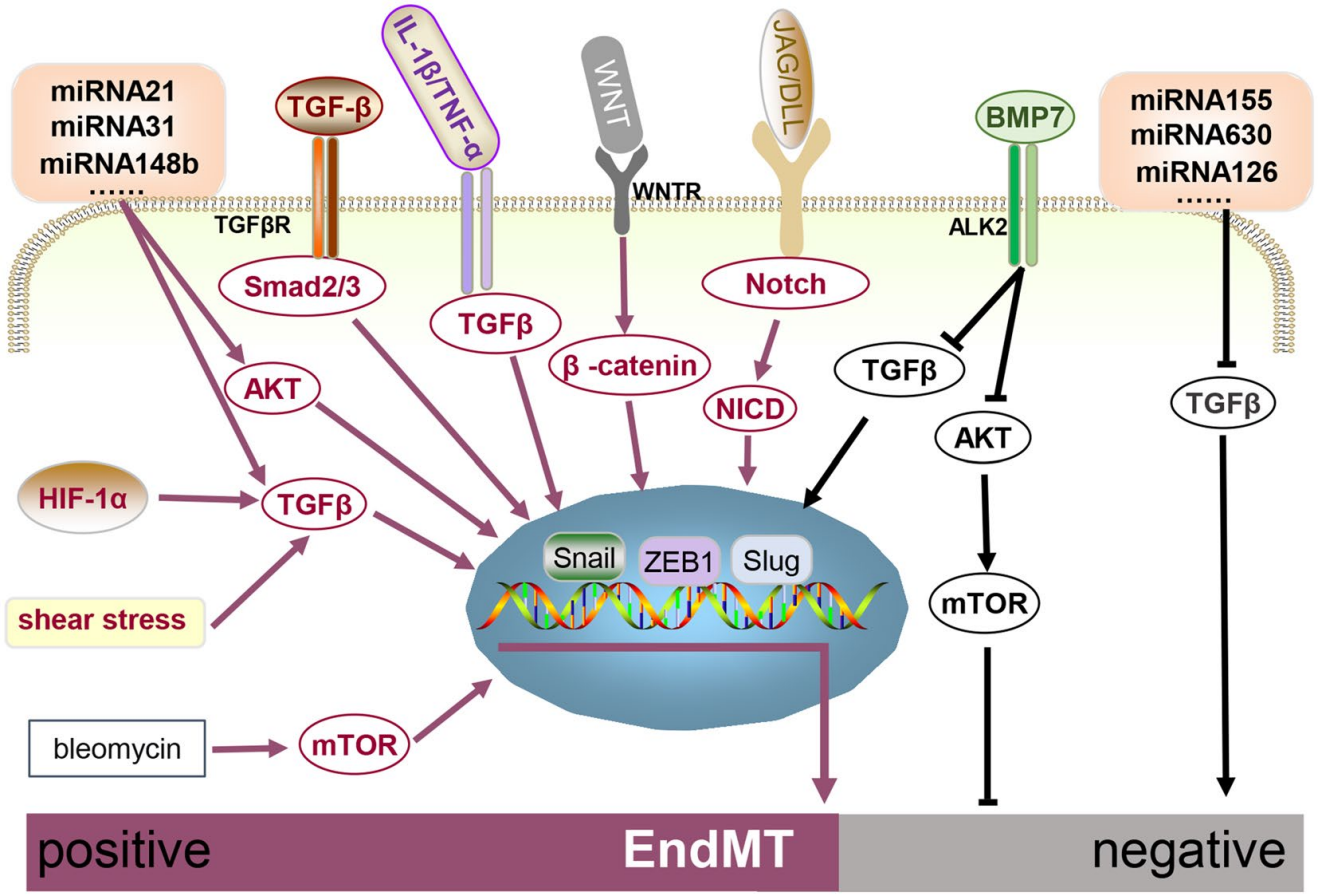
A schematic illustration of the signaling pathways governing EndMT. Various mechanisms regulate EndMT, the classic pathway is the TGF-β pathway, which regulates EndMT through Smad2/3 and transcription factors such as Snail and ZEB1. Multiple stimuli, including hypoxia, low shear, miRNA21, and inflammatory factors such as IL-1β and TNF-α, can act on TGF-β or act synergistically to promote EndMT. BMP7 inhibits EndMT by inhibiting TGF-β or AKT/mTOR. Some miRNAs exert inhibitory effects on EndMT

EndMT is a highly intricate process governed by diverse mechanisms that vary according to specific pathophysiological contexts. Although substantial research has been conducted on the epigenetic and genetic regulatory mechanisms of EndMT, the precise molecular alterations involved have not yet been fully elucidated. Here, we elaborate on the factors that moderate EndMT (Fig. 3).

### TGF-β pathway

TGF-β is recognized for its crucial function in preserving intercellular homeostasis in particular settings [40]. TGF-β is a major driver of EndMT during development and in the pathological environment of adulthood and can act through Smad-dependent and Smad-independent signaling pathways [41, 42].

Ma et al. revealed that TGF-β stimulates the expression of the EndMT transcription factors Snail and Slug, resulting in EndMT in mouse pancreatic microvascular ECs [43]. Moreover, TGF-β1 promotes EndMT by activating the Smad2/3-Snail signaling pathway in a mouse model of cardiac fibrosis [44]. The severity of human coronary artery disease is associated with reduced MAPK7 expression and elevated miR-374b expression. TGF-β-induced microRNA-374b promotes EndMT by inhibiting MAPK7 expression in ECs [45]. TGF-β signaling also reduces intercellular contacts and promotes angiogenesis [46], suggesting that TGF-β-stimulated ECs behavior is associated with EndMT, vascular permeability, and angiogenesis [47]. Studies have shown that knockdown or knockout of TGF-β signaling-related genes, such as Smad2, Smad3, and TGFBR2, can block EndMT [48].

Other TGF-β family members, such as BMPs, intricately regulate TGF-β signaling [49]. Among them, BMP7 inhibits EndMT by activating ALK2 and its associated Smad1/5/8 pathway [38]. Shen et al. reported that BMP7 reduces TGF-β-induced EndMT in HUVECs and mouse models of systemic sclerosis via the Akt/mTOR/p70S6K signaling pathway [50]. When BMPR2 is absent in ECs, the formation of a hybrid BMPR1/TGFβRI-type/TGFβRII-type heterodimer complex is favored, leading to canonical TGF-β signaling and increased EndMT in pulmonary arterial hypertension [51]. Thus, researchers have referred to endothelial BMPR2 as the gatekeeper that maintains the dynamic balance of BMP/TGF-β signaling.

### Notch signaling pathway

Several studies have shown that the Notch signaling pathway is key in EndMT. Notch signaling is initiated by ligand binding, which triggers cleavage of the transmembrane receptor, releasing the Notch intracellular structural domain (NICD), which is subsequently translocated into the nucleus to initiate transcription of targeted genes [52]. In the process of low oscillatory shear stress-induced EndMT, Li et al. reported that deletion of the Notch ligand DLL4 reduced the expression of Snail, a marker of EndMT, at atheroprone regions of arteries [53]. In human cerebral arteriovenous malformation nidus, oscillatory shear stress induces EndMT through Notch receptor activation, as indicated by increased Snail 1, Snail 2, and Slug expression. Blockade of the Notch signaling pathway effectively reduces the mesenchymal phenotype of ECs [54]. Furthermore, inhibiting JAG2/Notch signaling in JAG2-silenced HUVECs significantly reduces the expression of EndMT markers (Snail/Zeb1/N-cadherin/αSMA) [55]. In Kaposi sarcoma, KSHV induces EndMT through activation of the classical Notch signaling pathway and promotes the invasiveness and survival of infected ECs [33]. In breast cancer angiogenesis, TGF-β1 promotes EndMT through the TGF-β and Notch signaling pathways, in which Snail and Slug are key factors [56].

### Wnt signaling pathway

Wnt signaling has also shown to be important for EndMT. Once the Wnt signaling pathway is activated, β-catenin translocated to the nucleus and promotes LEF/TCF-mediated transcriptional regulation. After myocardial infarction, Wnt signaling is activated and EndMT is induced, in addition, activation of Wnt signaling with BIO induced the expression of EndMT associated genes Slug and αSMA, as well as the downregulation of CD31 in cultured ECs [57]. Another study reported that decreased plasma levels of sFRP3 post myocardial infarction, which activates the Wnt signaling pathway in sheep primary mitral valve ECs, allows TGF-β initiation EndMT [58]. Lipphardt et al. demonstrated that renal microvascular ECs acquired αSMA expression after application of Dickkopf-3 (DKK3), an agonist of the Wnt pathway, indicating that EndMT was undergone [59]. However, in aortic ECs, blocked of the Wnt signaling with Dkk-1 enhances EndMT and accompanied with tissue fibrosis [60]. In addition, Wnt/β-catenin pathway mediates the effects of miR-222 to block EndMT in diabetic mouse hearts, and thus miR-222 attenuates cardiac fibrosis [61]. Hence, the role of the Wnt signaling pathway in EndMT in different diseases still requires further investigation.

### Inflammation and oxidative stress

Proinflammatory molecules such as IL-1β [62], IL-6 [63], IFN-γ [64], and TNF-α [65] act synergistically with TGF-β to activate the expression of Snail and Slug, thereby stimulating EndMT. Matrix metalloproteinases (MMPs) also play a role in the inflammatory process by regulating various cytokines, chemokines, and extracellular matrix proteins [66]. In addition, HIF-1 has been shown to enhance TGF-β1 signaling by downregulating neprilysin, which leads to EndMT [67]. Yamashiro et al. reported that HIF-1α triggers partial EndMT and CD45 expression in human aortic ECs, subsequently promoting neointimal formation in ligated mouse carotid arteries via EndMT; notably, partial EndMT allows cells to preserve cell‒cell junctions during vascular remodeling [68].

### Epigenetic regulation of EndMT

Epigenetics has garnered significant recognition for its pivotal role in regulating gene expression without altering the underlying DNA sequence, primarily through mechanisms such as DNA methylation, histone modifications, and noncoding RNA regulation. Originally associated with cellular differentiation, epigenetics is now understood to be critically involved in a wide array of pathophysiological conditions, including EndMT.

#### DNA methylation

DNA methylation represents a ubiquitous and standard modification within eukaryotic cells, serving as the primary epigenetic mechanism for regulating gene expression in mammals. This modification predominantly occurs at cytosine‒phosphate‒guanine (CpG) islands in gene promoter regions, although various other methylation patterns exist. As a critical epigenetic process, DNA methylation plays a significant role in modulating gene activity, and genetic information can be transmitted to the DNA of offspring via the regulation of DNA methyltransferases (DNMTs) [69]. Tan et al. demonstrated that human coronary artery endothelial cells (HCAECs) undergo EndMT when exposed to elevated phosphate levels. Furthermore, they reported that EndMT is initiated through the recruitment of aberrantly phosphorylated DNMT1 to the RASAL1 CpG island promoter by histone deacetylase 2 (HDAC2), resulting in abnormal promoter methylation and transcriptional suppression. This, in turn, leads to increased Ras-GTP activity and the activation of common EndMT regulators, Twist and Snail [70]. Endothelial DNMT1 functions as a critical epigenetic regulator of hemodynamically induced EndMT, primarily through repressing ALDH2, ALDH3A1, and ALDH6A1 expression [71]. Methyl-CpG binding protein 2 (MeCP2) expression is upregulated during TGF-β-induced EndMT, and silencing MeCP2 effectively inhibits the induction of EndMT. Mechanistically, MeCP2 overexpression in ECs led to increased methylation of CpG islands within the BMP7 promoter [72].

#### Histone modification

Posttranslational modifications of histones within chromatin are pivotal in regulating gene expression. Research indicates that HDACs are involved in various posttranslational modifications, such as methylation, acetylation, glycosylation, S-nitrosylation, sumoylation, ubiquitination, and phosphorylation. Among these, histone methylation and acetylation have been extensively studied [73].

Histone methylation represents a critical posttranscriptional modification, predominantly regulated by histone methyltransferases. These enzymes facilitate the transfer of methyl groups to lysine residues on histones, utilizing S-adenosyl methionine as a methyl donor. Methylation occurs at the nitrogen atom of the side chains of lysine and arginine residues. Notable examples of histone methylation include H3 lysine 4 (H3K4), histone H3 lysine 9 (H3K9), and histone H3 lysine 27 (H3K27) methylation. Neumann et al. reported that EndMT is correlated with alterations in H3K4me3 methylation of two key EndMT-associated genes. This process is likely mediated through an interaction between the long noncoding antisense transcript of GATA6 and the epigenetic regulator LOXL2 [74]. Enhancer of zeste homolog 2 (EZH2) serves as the principal methyltransferase within polycomb repressive complex 2 (PRC2). The SET domain of EZH2 facilitates the trimethylation of histone H3 at lysine 27 (H3K27me3), thereby sustaining the repressive state of downstream target genes implicated in EndMT [75]. A previous study demonstrated that Twist is significantly upregulated during TGF-β-induced EndMT. At the VE-cadherin promoter, there was an increase in H3K9 methylation and a concomitant decrease in H3K4 and H3K56 acetylation. Twist was found to form a functional complex with H3K9 methyltransferase and HDAC, mediating the transcriptional repression of VE-cadherin [76].

Histone acetylation predominantly occurs at the highly conserved lysine residues at the N-terminus of histones H3 and H4. This process is orchestrated by histone acetyltransferases (HATs) and HDACs. Histones contain numerous lysine residues subject to acetylation, including but not limited to H3K4, H3K9, and H3K27. Acetylation of these lysine residues modulates protein function by inducing structural alterations or modifying the protein’s affinity for various binding partners [69]. One study demonstrated that deacetylation, particularly that mediated by HDAC9, plays a significant role in both EndMT and atherosclerosis [77].

### Noncoding RNAs

Noncoding RNAs (ncRNAs) encompass diverse RNA molecules that are not translated into proteins. This category includes ribosomal RNAs (rRNAs), transfer RNAs (tRNAs), small nuclear RNAs (snRNAs), small nucleolar RNAs (snoRNAs), microRNAs (miRNAs), and messenger RNAs (mRNAs), among others. These ncRNAs are characterized by a wide range of known and yet-to-be-discovered functions [69].

#### MiRNA

miRNAs are involved in the posttranscriptional regulation of gene expression by tissue-specific epigenetic modifications that modulate their activity. miRNAs are differentially modulated during TGF-β-induced EndMT; some act as EndMT inducers, whereas others appear to have inhibitory effects on EndMT. Kumarswamy et al. reported that miR-21 partially regulates TGF-β-mediated EndMT in cardiac ECs via a PTEN/Akt-dependent pathway [78]. Loss of function of miR-148b promotes EndMT by increasing the expression of TGF-β2 and Smad2, which in turn acquire a mesenchymal phenotype in ECs in a mouse skin wound healing model [79]. Global transcriptome analysis revealed that miR-31 is required for TGF-β-driven EndMT, not only upregulating the expression of mesenchymal markers but also increasing the expression of many chemokines and cytokines involved in inflammation [80]. Sun et al. reported that miR-630 negatively regulates EndMT by downregulating Slug in traumatic heterotopic ossification [81]. Moreover, the overexpression of miR-155 inhibits RhoA signaling in ECs and suppresses TGF-β-induced EndMT, which maintains ECs characteristics and blocks the acquisition of a mesenchymal phenotype [82]. In addition, Xiang and colleagues reported that miR-145 rescues CD31 and VE-cadherin expression downregulated by TGF-β and inhibits mesenchymal markers, confirming the inhibitory role of miR-145 in TGF-β-induced EndMT in endothelial progenitor cells [83]. Similarly, miR-126 interacts with PIK3R2 to inhibit TGF-β-induced EndMT in rat bone marrow-derived endothelial progenitor cells [84]. Zhu et al. revealed that miR-302c in ECs suppresses EndMT by directly binding to the 3’ UTR of MTDH and inhibiting its expression in hepatocellular carcinoma [85]. miR-20a targets various genes in the TGF-β signaling pathway, thus negatively regulating EndMT, while miR-132-3p has been identified as a novel EndMT regulator in the aorta through targeting of KLF7 [86].

#### Long non-coding RNAs (LncRNAs)

Long noncoding RNAs (lncRNAs) are nucleotide sequences exceeding 200 base pairs that are not translated into proteins. LncRNAs modulate gene expression patterns by influencing chromatin architecture and DNA accessibility through various molecular mechanisms, including signaling, baiting, guiding, and scaffolding [87]. They exhibit significant functional specificity by engaging in and modulating diverse cellular processes, including EndMT.

The lncRNA MAGOH-DT is a key mediator in TNF-α/high glucose-induced EndMT in arteriosclerosis obliterans. Inhibition of MAGOH-DT effectively reduces EndMT processes without changing TGF-β2 expression [88]. Yu et al. demonstrated the critical role of lncRNA H19 in EndMT under hypoxic conditions. Specifically, lncRNA H19 knockdown notably reduced hypoxia-induced EndMT through the let-7 g/TGF-βR1 axis in hypoxic pulmonary hypertension [89]. LncRNA DANCR deficiency promoted high glucose-mediated EndMT in cardiac microvascular cells via the FoxO1/DDAH1/ADMA pathway [90]. Similarly, lncRNA SNHG7 overexpression suppresses high glucose-induced EndMT in human retinal microvascular ECs [91]. LncRNA LINC00961 knockdown attenuated EndMT induced by TGF-β through the PTEN-PI3K-AKT pathway [92], while SENCR silencing aggravated the EndMT process and inhibited tube formation in ECs [93].

### Other factors

Other positive regulators of EndMT include bleomycin, which promotes Slug-dependent EndMT by activating the mTOR signaling pathway in long-term-treated HUVECs [94]. Hydrogen sulfide can ameliorate EndMT induced by endoplasmic reticulum stress via the Src signaling pathway, as manifested by the downregulation of mesenchymal marker expression, upregulation of endothelial marker expression, and restoration of the migratory capacity of HUVECs [95]. High concentrations of safrole oxide induce EndMT in HUVECs via the ATF4/p75NTR/IL-8 pathway [96]. In addition, using a mouse model with conditional deletion of endothelial carnitine palmitoyltransferase I, Xiong et al. demonstrated that endothelial fatty acid oxidation (FAO) is also a crucial factor in modulating EndMT and revealed that FAO regulates embryonic EndMT by altering intracellular acetyl-CoA levels I [97].

## Physiological functions of EndMT in the process of vascular development

EndMT plays a critical role in embryonic development. In the developing vertebrate embryo, the ECs of the heart initially exhibit an endothelial phenotype, expressing VE-cadherin and CD31, followed by loss of cell-cell contacts, cell migration, and upregulation of α-SMA expression, generating cardiac cushioning cells through the EnMT process to form the mesenchymal component of the septum and valves [98]. Romano showed that TGF-β2 stimulated EndMT and promoted endocardial cushion and heart development through chicken models [99]. During aortic development in the chick embryo, using structural and ultrastructural study methods, Arciniegas et al. showed that ECs of the aortic wall were activated and transformed into mesenchymal cells, which progressively combined and organized to form smooth muscle cells in chick embryos from day 7 to day 18 of development [100]. Quail embryonic dorsal aortic ECs transdifferentiate into mesenchymal cells that express α-SMA [101]. Thus, EndMT contributes to the formation of the endocardial cushion, that subsequently promotes the formation of the atrial septum, ventricular septum, and heart valves. Furthermore, EndMT is also involved in the maturation of the pulmonary arteries. During the advanced stages of chick embryonic development, ECs in the pulmonary artery explants of 10 ~ 11-day-old chicken embryos acquired mesenchymal characteristics, and some embryonic pulmonary ECs lost the expression of vWF and obtained α-SMA expression, contributing to intimal thickening and pulmonary artery remodeling [102]. Notably, EndMT also promotes early postnatal retinal angiogenesis, which is a transition modulated by VEGF signaling [103].

## Pathological function of EndMT in cerebrovascular disease

While EndMT is a critical process in embryonic development, studies report that EndMT is also notably associated with some pathogenetic circumstances, such as vascular disorders [104].

### Atherosclerosis

Atherosclerosis is a pathological condition characterized by the accumulation of plaques within arterial walls. This process is frequently initiated by hypercholesterolemia, an elevated concentration of plasma cholesterol, which alters the permeability of endothelial cell membranes, facilitating the infiltration of low-density lipoprotein (LDL) particles into the arterial intima. Subsequently, monocytes are recruited to the subendothelial space, differentiating into foam cells, exacerbating cholesterol accumulation, and promoting the differentiation of vascular smooth muscle cells. Endothelial dysfunction plays a critical role in the onset and progression of atherosclerosis. In recent years, the role of EndMT in atherosclerosis has been extensively studied in mouse models of atherosclerosis and validated in human atherosclerotic plaques [18, 29]. Chen et al. revealed that EndMT, promoted by HDAC3 overexpression via the modulation of inflammatory cytokines, contributes to the progressive growth of atherosclerotic plaques in ApoE^−/−^ mice [105]. Using an endothelial-specific Hdac9 knockout mouse model, Lecce et al. found that both EndMT and atherosclerotic burden were reduced and enhanced plaque stability was achieved, similarly, class IIa HDAC inhibition has the same effects [77]. Immunofluorescence staining showed that thoracic aortic plaques express the fibroblast-specific marker Fap, confirming the occurrence of EndMT in atherosclerosis and that EndMT-derived fibroblast-like cells play important roles in plaque destabilization [29]. In a mouse model of atherosclerosis, Chen et al. reported that EndMT is induced when atherosclerosis initiates, promoting plaque growth and neointimal expansion by forming new mesenchymal cells [18]. In addition, the role of lncRNAs in EndMT-regulated atherosclerosis was also studied. MALAT1 overexpression promoted ox-LDL-induced EndMT in HUVECs, contributing to atherosclerosis [106]. Clock expression was lower in carotid plaque samples from patients who underwent carotid endarterectomy. Furthermore, using a mouse model of carotid atherosclerosis, Tang et al. reported that Clock mutation activated the IRE1α‒XBP1 axis, promoting EndMT in ECs under disturbed flow, thereby aggravating carotid artery stenosis [107]. Obviously, more research is needed to resolve the contribution of EndMT to atherosclerosis in different models.

Given the positive role of EndMT in atherosclerosis discussed above, targeting EndMT to alleviate atherosclerosis is an effective approach. Diao et al. reported that the Chinese Fufang Zhenzhu Tiaozhi capsule mitigates atherosclerosis by inhibiting EndMT through the Akt1/β-catenin signaling pathway, improving endothelial dysfunction [108]. Tenascin-X, an extracellular matrix protein, inhibits EndMT by preventing the activity of TGF-β and thereby interfering with the progression of atherosclerosis [109]. In addition, Zhang et al. proposed three approaches to alleviate atherosclerosis via EndMT-derived MSCs [110]: inducing MSC differentiation into other cell types to attenuate atherosclerosis; reprogramming EndMT-derived MSCs in atherosclerosis into VECs using chemical reprogramming; and maintaining the properties of EndMT-derived MSCs in atherosclerosis. Given that atherosclerosis is a chronic disease, long-term inhibition of EndMT may be required to achieve favorable therapeutic effects on atherosclerosis.

### Ischemia stroke

Stroke is a devastating neurological disease accompanied by high mortality and disability [111, 112]. Using inducible Cdh5-Cre lineage-tracking mice, Chen et al. demonstrated that ECs in the brain undergo EndMT after MCAO through activation of the let-7i/TGF-βR1 double-negative feedback loop [113]. Knocking out MMP-3 reduces the expression of EndMT-related genes, including Snail, Twist, and TGF-β1, in the brain during the subacute stroke phase [114]. A recent study reported that PATJ was downregulated after ischemic stroke, and the depletion of PATJ exhibited hallmarks of EndMT, which is beneficial for stroke recovery [115]. In addition, circUCK2/HECTD1 overexpression in the ECs of tMCAO mouse brain tissues suppressed mesenchymal cell marker expression and reduced Evans blue staining, suggesting that circUCK2 protects the BBB by inhibiting EndMT [116]. So, targeting EndMT may hold therapeutic promise for stroke by preserving the structure and function of the BBB. Similarly, the circular RNA DLGAP4, downregulated in the plasma of acute ischemic stroke patients and tMCAO mice, can inhibit EndMT by targeting miR-143 in the ECs of tMCAO mice [117]. Oxygen glucose deprivation/reperfusion induces EndMT, while circHECTD1 knockdown notably suppresses this phenomenon via the miR-335/NOTCH2 axis in ischemic stroke [118]. At present, there is no clear evidence to confirm the therapeutic role of EndMT in ischemic stroke. Based on the above studies, under the premise that EndMT inhibition is beneficial to ischemia stroke, targeting different aspects of the MMP-3, PATJ, TGF-β, or Notch signaling pathways may help to attenuate EndMT, with subsequent reductions in ischemia stroke.

### Aneurysm

Aneurysm is a permanent swelling disease due to weakness in the arterial walls. Aneurysms can form anywhere, but the most common and troublesome sites for aneurysms are the cerebral arteries, the aorta, and the great arteries of the heart, where aneurysm rupture is often catastrophic when wall stresses exceed the pressure that the aneurysmal aorta can withstand. ECs are critical in aneurysm development. Several studies have reported that miRNAs regulate the vascular cell phenotype in genetic thoracic aortic aneurysms (TAAs). miR-632 upregulation induced by TGF-β1 in Marfan syndrome TAAs inhibits DNAJB6 expression, activating Wnt/β-catenin signaling to exacerbate EndMT [119]. SPP1 is an essential component of EndMT associated with developing degenerative ascending aortic aneurysms; specifically, SPP1 expression is upregulated in the aortic intima-media and correlates with an inflammatory expression profile [120].

Furthermore, reducing wall shear stress (WSS) can activate Ang II and downregulate miR-29 expression, promoting EndMT by activating the TGFBR2/Smad3 axis and accelerating the occurrence and progression of intracranial aneurysms [121]. Therefore, indirect inhibition of EndMT by Ang II receptor antagonists may be a potential target in the treatment of intracranial aneurysms. Arterial bifurcations are prone to aneurysms, and high WSS loading is necessary for initial aneurysm development [122]. Through analyzing EndMT-related genes in unruptured aneurysms, ruptured aneurysms, and healthy samples, Jiang et al. revealed that SPARC and FN1 were good predictors of intracranial aneurysms, and that these genes can be used as a screening method for identifying asymptomatic patients with ruptured aneurysms to reduce rupture of the aneurysms [123]. Indeed, most of the mechanisms of EndMT in aneurysms remain unclear and still require further in-depth studies.

### Arteriovenous malformations

Arteriovenous malformation (AVM) is a congenital vascular dysplasia of the central nervous system that can occur in various parts of the brain. It is characterized by a lack of direct communication between arterioles and veins without capillary beds. It is a significant cause of intracranial hemorrhage in children and young adults. Recent studies have demonstrated that EndMT is crucial in aberrant vessel development and pathology. A comparison of mesenchymal protein expression in cerebral AVM samples and control samples revealed that oscillatory shear stress induces EndMT, which requires Notch receptor activation. This effect was blocked when the Notch signaling pathway was inhibited by DAPT, even in the presence of oscillatory flow [54].

Similarly, Shoemaker et al. reported that tissue from human brain AVMs expressed higher levels of KLF4, SNAI1, Slug, TWIST1, ACTA2, vimentin, and S100A4, EndMT-associated transcription factors and markers, along with robust collagen deposition, than those observed in normal brain, indicating the role of EndMT in AVM disease [124]. Since KRAS mutations are found in brain AVMs, a recent study reported that exosomal miR-3131 promotes the EndMT process in KRAS-mutant HUVECs by targeting PICK1, suggesting that miR-3131 might be a potential biomarker and therapeutic target in brain AVMs with KRAS mutations [125]. Furthermore, Xu et al. reported that HUVECs overexpressing KRAS^G12D^ undergo EndMT by activating the TGFβ-BMP-SMAD4 signaling pathway. This process can be reversed by lovastatin, a lipid-lowering drug, by inhibiting the TGF-β/BMP pathway and SMAD4 acetylation [126]. Shoemaker et al. further verified that SMAD4-dependent signaling is not the primary mechanism in AVMs [127], therefore it may be involved in other signaling pathways. RNA-seq data from 66 AVM nidus and control vessels from 7 non-AVM epilepsy patients revealed lower expression of ECs markers in AVWs. Notably, single-cell RNA-seq revealed mesenchymal marker expression in contrast to that in ECs in non-AVM human brain tissue, confirming the occurrence of EndMT in human AVMs. In addition, investigators reported that lovastatin alleviates EndMT induced by KRAS mutation in both in vitro and ex vivo trials, providing new preclinical evidence [128]. U0126, a selective inhibitor of MEK1/2, reversed EndMT in HUVECs, suggesting the importance of MAPK-ERK signaling in mediating KRAS mutation-induced EndMT [126, 129], so that inhibition of EndMT via the MEK/ERK blockade could be a mechanism of considerable benefit in AVMs treatment. Accordingly, Sox2 signaling interacted with JMJD5 to induce EndMT in ECs, resulting in lumen disorder in cerebral AVMs [130]. Single-cell mRNA sequencing (scRNAseq) of brain arteriovenous malformations indicated that gene set enrichment analysis confirms pathogenic pathways, including angiogenesis, inflammation, and EMT, which are enriched in the AVM nidus 2 endothelium [131].

While the link between AVMs and EndMT has only been recognized in the last two years, experimental evidence is still preliminary, the exact molecular mechanisms of EndMT in AVMs are largely unknown. Further experimental evidence is needed to support this in clinical translation. Hence, investigating the molecular regulatory mechanisms of EndMT and identifying specific EndMT inhibitors will be of great benefit in the development of AVM patients.

### In-stent stenosis

In-stent stenosis (ISR) is a complex disease process in which stent placement (bare metal stents, drug-eluting stents) during vascular interventions causes vascular injury, leading to fibroblast proliferation and neointimal hyperplasia, resulting in luminal stenosis [132]. It is widely accepted that intimal hyperplasia with SMCs proliferation as the core is the primary pathological process of in-stent restenosis. While drug-eluting stents developed with sirolimus and paclitaxel as the target can reduce the incidence of in-stent restenosis to a certain extent, the incidence of late in-stent restenosis is still as high as 10% [133]. In studies with poly-L-lactic acid-induced ISR, Hou et al. reported that lactic acid triggered EndMT through TGF-β1 signaling, further leading to vascular fibrosis and contributing to severe ISR [134]. It can be speculated that lactic acid and TGF-β1 signaling could be potential targets in EndMT-mediated in-stent restenosis. This provides a new targeted therapeutic strategy for the clinical treatment of ISR, but more experimental evidence and clinical data are needed.

### Moyamoya disease

Moyamoya disease (MMD) is a rare, chronic, progressive cerebrovascular disorder characterized by obscure etiological factors and pathophysiological mechanisms. Increasing evidence indicates that MMD is predominantly described as a proliferative disorder of the intimal layer [135, 136]. The reduced expression of endothelial markers in ECs associated with MMD, coupled with the upregulation of mesenchymal markers in the intima and arachnoid membrane, suggests the potential involvement of EndMT in the pathogenesis of MMD [137, 138]. A prior investigation demonstrated positive αSMA staining in the occlusive arteries of patients with MMD, suggesting the occurrence of EndMT under these conditions [139]. Furthermore, the downregulation and delocalization of CD31 in an EC model of MMD indicate that compromised cerebral endothelial integrity may represent an early pathological mechanism contributing to the development of MMD [138]. A recent study demonstrated that the pathological characteristics of MMD are largely influenced by naive ECs, as evidenced by a comparative analysis of transcriptomic profiles between healthy controls and individuals with MMD [140]. TGFβ1, a potent inducer of EndMT, was significantly upregulated in MMD and correlated with the development of collateral vessels [141]. Further research on EndMT-related genes (ERGs) in MMD identified ERGs that were differentially expressed between MMD patients and controls. Following validation in the testing set, four ERGs—CCL21, CEBPA, KRT18, and TNFRSF11A—were identified as the principal MMD-related hub ERGs. Importantly, in vitro experiments demonstrated that these ERGs could promote proliferation, migration, and EndMT [142].

Given their crucial role in inducing and maintaining EndMT, the TGF-β signaling pathway and ERGs may be obvious targets for EndMT. These findings emphasize the critical involvement of EndMT in the pathogenesis of MMD, underscoring the need to investigate the molecular characteristics associated with EndMT to facilitate the development of innovative diagnostic biomarkers, therapeutic targets, and treatment strategies for MMD.

### Dural arteriovenous fistula

Dural arteriovenous fistulas (DAVFs) represent an infrequent yet extensively documented etiology of intracranial hemorrhage [143]. Arteriovenous fistulas were investigated using macroscopic and histological analyses, employing hematoxylin, eosin, and safran (HES), as well as orcein stains. In most vessels examined, the venous wall was characterized by ECs situated on layers of smooth muscle, delineated by an elastic lamina, emphasizing the critical involvement of EndMT in the pathogenesis of DAVF [144, 145]. An animal model study demonstrated an association between DAVFs and vascular endothelial growth factor (VEGF) expression [146]. VEGF expression is stimulated by tissue hypoxia, which may be caused by venous hypertension and increased internal pressure within blood vessels [147]. In a cohort of ten patients diagnosed with DAVF, plasma levels of VEGF were measured before and after endovascular treatment. The findings revealed that 80% of patients exhibited elevated plasma VEGF levels prior to the intervention [148]. Due to the frequent paucity of histopathological data on DAVFs, there is potential for controversy in developing novel diagnostic biomarkers, therapeutic targets, and treatment strategies. Additional studies are still needed to reveal the regulatory mechanisms and therapeutic targets of EndMT in DAVFs.

### Cerebral venous sinus thrombosis

Cerebral venous sinus thrombosis, a primary form of venous stroke, represents a distinct subtype characterized by the disruption of venous blood flow resulting from thrombosis in venous vessels. This condition accounts for 0.5–1% of all strokes, yet it constitutes 14–20% of strokes occurring in young adults [149]. Virchow’s triad elucidates the role of blood hypercoagulability, blood flow dynamics, and endothelial damage in the pathogenesis of cerebral venous thrombosis [150]. Our study conducted a detailed analysis of pathomorphological alterations in the ECs of the venous sinus wall in a rat model of venous hypertension, utilizing transmission electron microscopy. Compared with the sham control group, the venous hypertension group presented reduced tight junctions between ECs in the superior sagittal sinus. Importantly, the expression of von Willebrand factor (vWF) and coagulation factor VIII (F8) in the complement and coagulation cascades, along with Fgg and F2 in platelet activation, was increased in the cerebral venous sinuses of a rat model of venous hypertension [151]. It has been proposed that damage to ECs reduces the phenotype characterized by the number of tight junctions, ultimately increasing the risk of cerebral venous thrombosis [152]. Therefore, platelet activation and the complement and coagulation cascades might be potential targets for the prevention of cerebral venous sinus thrombosis, in which EndMT exerts significant effects.

## Conclusions and perspectives

EndMT, EMT, and MET are conserved intact cellular processes throughout organism formation and pathological end-organ diseases, where EndMT represents the prototypical plasticity of ECs. It plays a significant role in physiological processes, such as vascular and embryonic development, and is indispensable in pathological processes, such as cardiovascular diseases. Therefore, exploring the molecular mechanisms involved in EndMT is critical. Several studies have confirmed that targeted inhibition of EndMT can be used as a therapeutic route for various diseases (as discussed in the text). Still, no effective therapeutic strategy is available in the clinical setting. The primary challenge is the complexity of the EndMT regulatory network, and the mechanisms currently available to researchers are insufficient to identify effective EndMT inhibition targets. There is still a need to decipher the unknown EndMT regulatory network, especially the key differences between pathological and physiological EndMT, and to provide solid and reliable evidence to support the selection of EndMT targets. In addition, there is significant controversy over the assay results due to the use of different cellular markers, detection time points, and induction factors. To overcome these challenges, exploration of more refined EndMT regulatory mechanisms is needed to lay the foundation for subsequent clinical development.

## Data Availability

All data are included in the manuscript.

## Abbreviations

EndMT: endothelial–mesenchymal transition
EMT: epithelial–mesenchymal transition
ECs: endothelial cells
VECs: vascular endothelial cells
MSCs: mesenchymal stem cells
HUVECs: human umbilical vein endothelial cells
SMCs: smooth muscle cells
EPCs: endothelial progenitor cells
NICD: Notch intracellular structural domain
MMPs: Matrix metalloproteinases
CpG: cytosine‒phosphate‒guanine
DNMTs: DNA methyltransferases
HCAECs: human coronary artery endothelial cells
HDAC2: histone deacetylase 2
MeCP2: Methyl-CpG binding protein 2
H3K4: H3 lysine 4
H3K9: histone H3 lysine 9
H3K27: histone H3 lysine 27
EZH2: enhancer of zeste homolog 2
PRC2: polycomb repressive complex 2
H3K27me3: trimethylation of histone H3 at lysine 27
HATs: histone acetyltransferases
ncRNAs: Noncoding RNAs
rRNAs: ribosomal RNAs
tRNAs: transfer RNAs
snRNAs: small nuclear RNAs
snoRNAs: small nucleolar RNAs
miRNAs: microRNAs
mRNAs: messenger RNAs
lncRNAs: Long noncoding RNAs
LDL: low-density lipoprotein
TAAs: thoracic aortic aneurysms
WSS: wall shear stress
AVM: Arteriovenous malformation
ISR: in-stent stenosis
MMD: moyamoya disease
ERGs: EndMT-related genes
DAVFs: dural arteriovenous fistulas
HES: hematoxylin, eosin, and safran
VEGF: vascular endothelial growth factor
vWF: von Willebrand factor
F8: coagulation factor VIII
